# Unexpected Failure of Plate Osteosynthesis of a Proximal Humerus Fracture Following a Seizure Episode in the Early Postoperative Period

**DOI:** 10.7759/cureus.15909

**Published:** 2021-06-24

**Authors:** Arvind Kumar, Javed Jameel, Siddhartha Sinha, Sandeep Kumar, Yawar Haider

**Affiliations:** 1 Orthopaedics, Hamdard Institute of Medical Sciences and Research, New Delhi, IND

**Keywords:** failure, plate osteosynthesis, proximal humerus fractures, seizure, shoulder

## Abstract

Seizure disorders have been commonly linked with fractures and dislocations of the shoulder region. However, catastrophic failure of plate osteosynthesis of a proximal humerus fracture, following a seizure episode in an early postoperative period, has not been reported. We present a case report of a middle-aged male patient who was treated satisfactorily for a right proximal humerus fracture with plate osteosynthesis but experienced a seizure episode in the early postoperative period. The seizure episode resulted in a catastrophic failure of the plate fixation. The patient was then managed with plate and screw removal and revision fixation with a lightweight pin-based external fixator. Further, the functional outcomes were satisfactory. Extreme caution must be exercised to manage proximal humerus fractures in people with epilepsy concerning anticonvulsant drug compliance and osteoporosis.

## Introduction

Proximal humerus fractures, especially multifragmentary ones, often require surgical fixation for early rehabilitation and favorable function outcomes. The proximal humerus locking plates are the preferred devices for the fixation of these fractures [1]. These plates provide a stable construct and allow repair of the greater tuberosity and lesser tuberosity fragments that provide attachments to the rotator cuff muscles. The rehabilitation in patients operated with these devices is usually uneventful, and the fracture usually unites in few months [2]. Seizure-disorder patients often present with shoulder joint dislocations. The violent muscular contractions can bring sufficient force to dislocate the shoulder joint [3]. Even proximal humerus fractures have been reported with seizure disorders. However, implant fixation failure following proximal humerus fracture fixation in a seizure-disorder patient is an unforeseen challenging situation and has not been reported previously. We present a case report of a middle-aged male patient who was operated on for a right proximal humerus fracture with a proximal humerus locking plate but experienced a seizure episode in the postoperative period. The seizure episode resulted in a catastrophic failure of the plate fixation. The patient was then managed with plate and screw removal and revision fixation with a lightweight pin-based external fixator. Further, the functional outcomes were satisfactory.

## Case presentation

**Figure 1:**
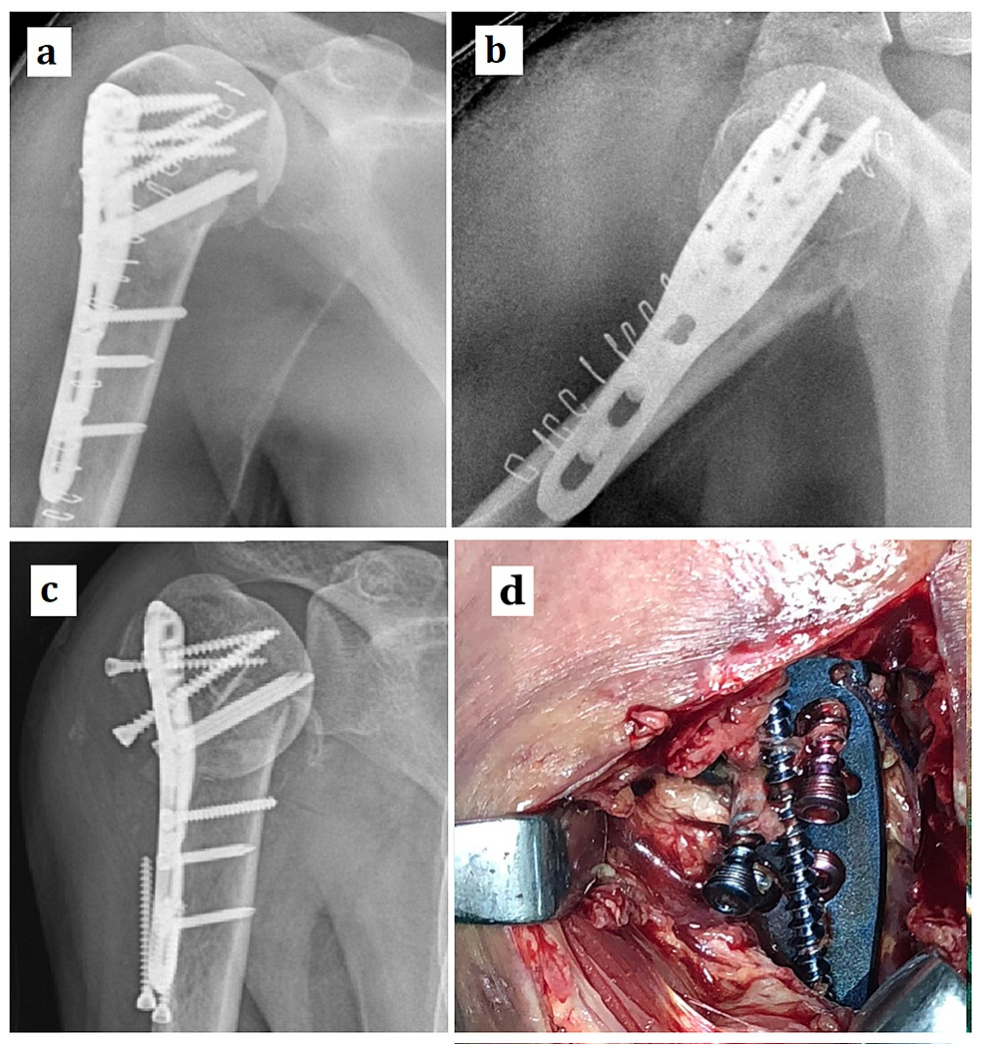
Postoperative radiographs, AP (a) and lateral (b), of a two-part proximal humerus fracture fixed with a proximal humerus locking plate have been shown. Following a seizure episode, the patient sustained a catastrophic fracture fixation failure with varus collapse of fracture (c). During the revision surgery, the intraoperative picture (d) shows the proximal locking cancellous screws migrated out of the proximal fragment. AP - anteroposterior

A 52-year-old male had presented to the emergency department with pain and swelling around the right shoulder region following a fall from stairs. The clinicoradiological evaluation suggested a two-part proximal humerus fracture. The patient had no other injuries, and systemic examination was normal. The patient was a known epileptic and was treated with regular anti-seizure medications. The fracture was managed operatively with an open reduction and internal fixation using a titanium proximal humerus locking plate (Figures 1a, 1b). The standard deltopectoral approach was used for fracture exposure. The bone was osteoporotic and the screw purchase is an obvious issue in such fractures. We used specially designed locking head cancellous screws for the humeral head fragment fixation. These screws combine the advantage of cancellous screws, which offer better bone purchase, and the locking head helps in angular stable construct for the patients with such osteoporotic bone. The perioperative period was uneventful. Osteoporotic management in form of calcium and vitamin D supplementation was started right from the admission and was maintained throughout the follow-up. Postoperatively, the patient was kept on a guarded mobilization program because of the poor bone quality intraoperatively. The patient was discharged on the third postoperative day and was called for suture removal after two weeks of surgery. The patient underwent suture removal, and the postoperative wound healed completely at the end of the second week. After the third postoperative week, the patient sustained a seizure episode. Non-compliance with antiepileptic medications was the probable cause for the seizure episode. Following the violent contractions of the right arm at the time of seizure, the patient had redeveloped a painful swelling around the right shoulder. Clinicoradiological evaluation revealed a catastrophic failure of the fracture fixation. The proximal screws had migrated to the deltoid insertion region, and the fracture collapsed in varus (Figures 1c, 1d). The violent abduction of the shoulder probably surpassed the fixation strength leading to such a failure.

**Figure 2:**
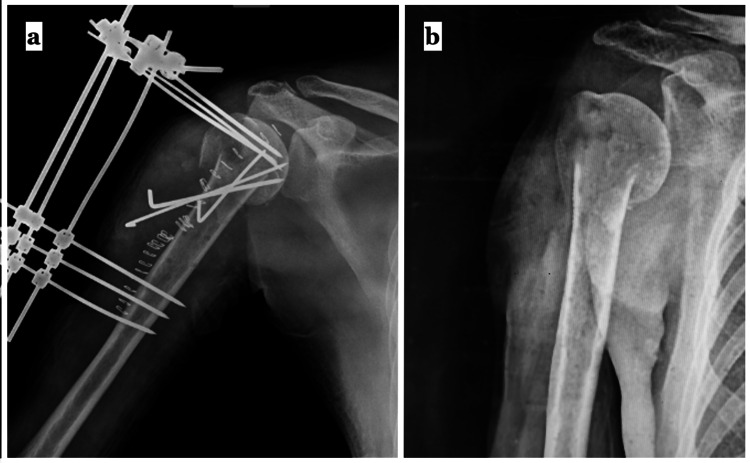
(a) A simple pin-based external fixator was used to salvage the implant failure following the seizure episode. (b) Follow-up radiograph at two months after the second surgery.

Under antiepileptics cover, the patient was re-operated, and the same deltopectoral interval was used. The plate and migrated screws were removed. The bone was osteoporotic, and a major segment of the fracture margins was already damaged. We docked the distal fragment (shaft fragment) into the humeral head, and a temporary reduction was achieved. A simple pin-based external fixator was then used to span the fracture while additional K wires held the humeral head to the humeral shaft (Figures 2a, 2b). The reduction was secured under direct vision. The wound was then closed in layers. Within two weeks, the wound healed satisfactorily. The fracture union was achieved in two months. The shoulder’s complete range of motion was restored within three months of the second surgery, and the patient did not face any limitations in daily activities. Strict compliance to the antiepileptic medications was ensured throughout the follow-up.

## Discussion

Seizure episodes are a common cause of fracture-dislocations of the shoulder joint [4]. Most of the reported shoulder injuries following a seizure disorder are acute dislocations and rarely fractures [5-7]. The current case report suggests that such forces can be strong enough to cause fracture fixation failure, even with a strong locking plate construct. Although it was known that the patient had a history of seizure disorder, it was not a contraindication for osteosynthesis. The patient had also resumed gentle range of motion exercises postoperatively, and timely sutures removal was done. Thus, a premature failure of fixation was not anticipated in this case. The pin-based fixator provided a simple alternative for such a case scenario, considering that the fixator will migrate only outwards in a similar unforeseen complication.

Classically the shoulder injuries in seizure episodes mainly constitute dislocations, which result from the persistent tonic force of the muscles around the shoulder [8]. Both anterior and posterior dislocations have been reported with variable incidence. One of the risk factors for shoulder injuries in epileptic patients is the non-compliance or reluctance to continue anticonvulsive medications [9]. Therefore, psychological assessment should be performed to predict compliance with anticonvulsive treatment. There are high chances that compliance-related information may get missed when the main focus is on injury management. However, that may add to the risk of future similar injuries. It may be stressed that anticonvulsant treatment aims to prevent future injuries and not solely the seizure episode. Therefore, perioperative counseling of epileptic patients regarding drug compliance is a must while managing bone and joint injuries.

During a seizure episode, the proposed mechanism of shoulder injury is strong adduction, internal rotation, and flexion forces [4,10]. The humeral head forcibly internally rotates by severe contraction leading to the dislocation of the head over the glenoid rim posteriorly. However, anterior dislocations have also been reported suggesting variable muscle forces during seizure episodes [11]. When the joint has already dislocated, or with a weak bone interface, a fracture of the proximal humerus may occur with aggregated forces from the muscles around the shoulder. As mentioned earlier in the current case study, the bone was osteoporotic, and therefore the weakest interface was probably at the fracture site rather than the joint. Therefore, the adduction, rotation, and flexion forces resulted in varus collapse as the fracture site. In addition, the screw pullout strength was reduced due to osteoporotic bone, and the violent muscle contraction resulted in the complete extrusion of the proximal screw down to the deltoid insertion. Considering the risk of poor bone purchase in osteoporotic bone, we had used specially designed locking head cancellous screws, which combine the benefit of the cancellous screw with a better bone purchase and locking screw heads that provide stable angle construct. In addition, osteoporotic management was started in form of calcium and vitamin D supplementation from the day of admission. We feel the intensity of the convulsion forces and the extent of osteoporosis could have contributed to the failure.

Implant failure in the early postoperative period following a humeral fracture fixation due to a seizure episode has not been reported in the past. With the unfamiliarity of such an injury pattern, it was difficult to proceed with revision plate fixation. In most situations with fracture proximal humerus following seizure episode, plate fixation is preferred. The situation in our case was complicated by post-seizure fixation failure and the osteoporotic bone. The fracture site was further comminuted due to injury forces. Based on our previous experience of using pin based external fixator for proximal humerus fracture treated in a closed manner, we felt the same would be optimum for this case. However, more evidence is needed to decide the optimum treatment modality in such patients. We feel a seizure-free period should be the aim, irrespective of the treatment modality. Therefore, specialist consultation should be sought during the patient follow-up.

## Conclusions

Extreme caution must be exercised to manage proximal humerus fractures in people with epilepsy. A seizure disorder in the postoperative period can result in a rare complication of fixation failure. Patients should undergo psychological counseling for strict compliance to antiepileptic medications to avoid such complications. In addition, osteoporotic management should be considered in all seizure-disorder patients as that is a potential risk factor for premature fixation failure.
